# Advances in mitochondrial-targeted colorectal cancer therapy: Mechanistic insights and clinical translation

**DOI:** 10.1016/j.isci.2025.114511

**Published:** 2025-12-27

**Authors:** Hao Che, Zhen-Jun Li, Ling Xu, Yang-Bing Du, Song-Ou Zhang, Xiao-Jiang Ying

**Affiliations:** 1 Department of Colorectal Surgery, Shaoxing People’s Hospital, Shaoxing, Zhejiang Province, China; 2 Ningbo University, School of Medicine, Ningbo, Zhejiang Province, China; 3 Department of Colorectal Surgery, Shaoxing People’s Hospital, Shaoxing, Zhejiang Province, China

**Keywords:** Immunology, Cancer, Biochemistry

## Abstract

Colorectal cancer (CRC) therapy is challenged by drug resistance and limited treatment efficacy. Mounting evidence now positions mitochondrial dysfunction as a central mediator of these challenges, making it a compelling therapeutic target. This review synthesizes findings demonstrating that targeting mitochondrial metabolism, apoptosis, dynamics, mitophagy, and intercellular transfer effectively overcomes chemoresistance and restores treatment sensitivity in CRC models. Key mechanisms include the reversal of the Warburg effect, reactivation of intrinsic apoptosis, and disruption of mitochondrial transfer. Clinically, mitochondrial-derived biomarkers, such as cell-free mtDNA, emerge as promising tools for non-invasive monitoring and prognosis. Furthermore, advancements in targeted delivery systems and supportive interventions such as exercise, are shown to enhance therapeutic efficacy and mitigate toxicity. We conclude that integrating mitochondrial-targeted strategies represents a transformative approach for CRC treatment, with future success hinging on overcoming delivery challenges and validating these strategies in personalized models.

## Introduction

Colorectal cancer (CRC), one of the most common digestive tract malignancies worldwide, presents an increasing burden. Geographically, the incidence rate is significantly higher in developed countries compared to developing countries, likely due to factors such as diet and lifestyle.^1^^,^^2^ In terms of disease characteristics, CRC exhibits a strong age correlation, with a marked increase in incidence among individuals over 50 years old. However, the incidence rate in younger populations has been rising in recent years, a trend that has garnered significant attention in the medical community.^3^^,^^4^ Currently, CRC treatment primarily follows a comprehensive model, with surgery as the main one. For early-stage patients, radical surgical resection is the preferred option, with a 5-year survival rate exceeding 90% after surgery.^5^ However, approximately 25% of patients present with distant metastasis at the time of initial diagnosis, and an additional 30%–50% of early-stage patients will experience recurrence and metastasis following surgery.^6^^,^^7^ For patients in advanced stages, systemic therapy is the primary treatment, with chemotherapy regimens such as FOLFOX and FOLFIRI being commonly used. While these regimens have improved survival to some extent, the overall efficacy remains suboptimal.^8^^,^^9^ The development of targeted therapies has offered new hope for CRC treatment. The application of anti-epidermal growth factor receptor (EGFR) therapy and anti-vascular endothelial growth factor therapy has significantly improved the prognosis for some patients.^10^ New therapeutic targets for CRC are constantly being discovered.^11^^,^^12^ In addition, many novel nanomaterials have also been applied to CRC research.^13^^,^^14^^,^^15^ However, these targeted drugs have obvious limitations.^16^ Although immunotherapy has shown considerable efficacy in microsatellite instability-high (MSI-H)/deficient mismatch repair patients, these patients represent only 5%–15% of the total CRC population, indicating the majority of patients do not benefit from this treatment.^17^^,^^18^^,^^19^ These challenges have prompted ongoing efforts to identify new therapeutic targets and strategies. Mitochondria have emerged as a promising area of research in CRC treatment. Increasing evidence suggests that mitochondrial dysfunction is closely related to the development and progression of CRC. The targeting of mitochondrial function heralds a paradigm shift for addressing current treatment bottlenecks.^20^^,^^21^

From the perspective of energy metabolism, mitochondrial dysfunction represents one of the most prominent metabolic hallmarks of CRC. The classic “Warburg effect” describes the phenomenon in which tumor cells preferentially rely on glycolysis for energy production, even under oxygen-rich conditions, rather than oxidative phosphorylation.^22^^,^^23^^,^^24^ This phenomenon is particularly prominent in CRC.^25^ CRC cells show significantly higher rates of glycolysis compared to normal intestinal epithelial cells, while mitochondrial oxidative phosphorylation function is relatively diminished.^26^ This metabolic reprogramming not only supplies the energy and biosynthetic precursors necessary for rapid tumor cell proliferation but also acidifies the TME by producing large amounts of lactate, thereby promoting tumor invasion and metastasis.^23^^,^^27^^,^^28^ Molecularly, this metabolic shift involves the abnormal expression of several key enzymes, including the upregulation of glycolytic enzymes such as hexokinase 2 (HK2) and pyruvate kinase M2 (PKM2), along with altered activities of enzymes in oxidative phosphorylation, such as mitochondrial complexes I, Ⅱ, and Ⅲ.^29^^,^^30^^,^^31^^,^^32^^,^^33^ Regarding apoptosis regulation, dysregulation of the mitochondrial pathway is a key mechanism by which CRC cells acquire resistance to apoptosis. Normally, mitochondria initiate the intrinsic apoptotic pathway by releasing pro-apoptotic factors such as cytochrome *c*.^34^ However, in CRC cells, this process is often disrupted by various factors. Studies have shown that CRC cells commonly overexpress anti-apoptotic proteins, such as B-cell lymphoma-2 (BCL-2), B-cell lymphoma-extra large (BCL-xL), and myeloid cell leukemia 1 (MCL-1), while downregulating or inhibiting the function of pro-apoptotic proteins such as Bcl-2-associated X protein (BAX) and Bcl-2 antagonist killer (BAK).^35^ Furthermore, the stability of mitochondrial membrane potential (ΔΨm) is closely associated with apoptosis resistance.^20^^,^^36^^,^^37^ Together, these alterations form the molecular basis by which CRC cells evade apoptosis and acquire chemoresistance. Dysregulated reactive oxygen species (ROS) homeostasis is another hallmark of mitochondrial dysfunction. Mitochondria are the primary source of intracellular ROS, and under normal conditions, ROS production and clearance maintain a dynamic equilibrium. However, in tumor cells, this balance is often disrupted. On one hand, moderate levels of ROS can promote tumor cell proliferation and survival by activating signaling pathways such as hypoxia-inducible factor 1-alpha (HIF-1α) and transcription factor NF-κB. On the other hand, excessive ROS can induce oxidative stress, leading to DNA damage and cell death.^38^^,^^39^ Dysfunction of the mitochondrial electron transport chain (ETC) in CRC cells often leads to abnormal ROS accumulation, which not only induces mtDNA mutations but may also drive tumor progression by activating multiple signaling pathways.^40^^,^^41^^,^^42^ The effects of ROS in CRC are concentration-dependent, providing valuable insights for the development of therapeutic strategies based on ROS regulation.^38^^,^^43^ Furthermore, recent studies have highlighted the important roles of mitochondrial dynamics and mitophagy in CRC. Mitochondrial dynamics describe the balance between morphology and function maintained through continuous fission and fusion.^44^^,^^45^ In CRC, this balance is frequently disrupted, resulting in enhanced fission and impaired fusion.^46^ This alteration not only affects mitochondrial energy metabolism but is also closely linked to tumor invasion and metastasis.^47^ Mitochondrial autophagy (mitophagy), a crucial quality control mechanism for selectively removing damaged mitochondria,^48^^,^^49^ is often impaired in CRC.^50^^,^^51^ Dysregulation of the PTEN-induced kinase 1 (PINK1)/Parkin pathway may lead to the accumulation of dysfunctional mitochondria, promoting tumor progression.^52^^,^^53^ These emerging findings significantly deepen our understanding of mitochondrial function in CRC and lay a theoretical foundation for the development of new therapeutic targets.

Mitochondrial-targeted therapeutic strategies present unique advantages and hold broad application potential in the treatment of CRC. Mitochondria are increasingly attracting researchers' attention as a potential therapeutic target for CRC.^54^^,^^55^ Their therapeutic value is primarily reflected in several key aspects. One significant benefit is their ability to overcome drug resistance. Alterations in mitochondrial metabolism have been identified as a key mechanism underlying chemotherapy resistance in CRC cells.^21^^,^^56^ For example, metformin, a mitochondrial complex I inhibitor, can reverse chemotherapy resistance by inhibiting oxidative phosphorylation. This mechanism restores the sensitivity of tumor cells to apoptosis and reduces the self-renewal capacity of cancer stem cells.^57^^,^^58^ Similarly, BH3-modified drugs, such as ABT-737, which target mitochondrial membrane permeability, can bypass drug-resistant mutations in the EGFR/RAS signaling pathway and directly activate the intrinsic apoptotic pathway.^59^^,^^60^ These findings offer new insights into addressing current drug resistance challenges in CRC treatment. Furthermore, mitochondrial-targeted drugs may exert synergistic effects when combined with traditional chemotherapy agents, potentially paving the way for the development of new combination therapies.^61^ Mitochondrial-targeted strategies also offer a distinct advantage in terms of their broad therapeutic potential. Unlike therapies that are limited to specific gene mutations, mitochondrial-targeted drugs may prove effective across multiple molecular subtypes of CRC. Mitochondrial dysfunction is a hallmark feature of all CRC types, and it is relatively independent of specific driver gene mutations. For example, mitochondrial ETC inhibitors have demonstrated inhibitory activity against multiple CRC subtypes, including microsatellite-stable CRC, in preclinical studies.^62^^,^^63^ This universality expands the patient population who could benefit from treatment and offers a new approach to addressing the limitations of current targeted therapies. Some mitochondrial-targeted drugs remain effective in CRC models that are resistant to both chemotherapy and targeted therapy, offering new hope for patients with advanced, refractory CRC.^64^^,^^65^ In addition, mitochondrial-targeted drugs show significant potential in combination therapies. Combining these agents with immunotherapy is a current focus of research. Studies have demonstrated that regulating mitochondrial metabolism can alter the immunosuppressive state of the TME. Inhibition of the mitochondrial pyruvate carrier can promote T cell infiltration and activation, thereby enhancing anti-tumor efficacy.^66^ Combining mitochondrial-targeted therapies with radiotherapy is another promising approach. Mitochondrial ROS modulators can significantly increase the sensitivity of tumor cells to radiation.^67^ Furthermore, combining mitochondrial-targeted drugs with traditional chemotherapy agents has also shown promising synergistic effects.^56^ These combination strategies not only enhance therapeutic efficacy but also allow for a reduction in the dosage of each drug, thereby minimizing toxic side effects.^56^ However, to ensure the success of these combinations, it is crucial to understand the mechanism of action of each drug in order to avoid potential antagonistic interactions. From a drug development perspective, the field of mitochondrial-targeted therapies is rapidly advancing. Several mitochondrial-targeted drugs, including metabolic regulators, apoptosis inducers, and ROS modulators, have already entered clinical trials.^68^^,^^69^^,^^70^ In parallel, the development of novel mitochondrial-targeted delivery systems, such as mitochondria-targeted nanoparticles, has greatly improved drug targeting and bioavailability.^71^^,^^72^ The search for reliable biomarkers to predict treatment efficacy is another key focus of ongoing research.^73^ Collectively, these advances are propelling mitochondrial-targeted therapies from the laboratory to clinical practice, offering new treatment options for patients with CRC. With continued research and technological progress, mitochondrial-targeted strategies are poised to become an integral component of comprehensive CRC treatment.

## Mechanisms of mitochondrial-targeted therapy

### Metabolic intervention strategies

**Figure 1:**
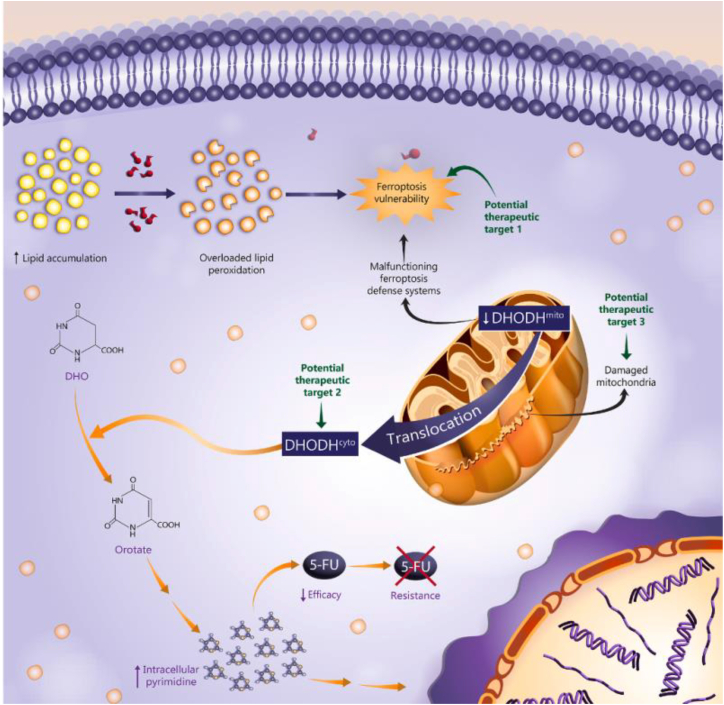
The proposed mechanism of DHODH in 5-FU-resistant CRC Reproduced with permission from ref.^64^ The susceptibility of 5-FU-resistant CRC cells to ferroptosis was increased due to lipid peroxidation overload, a consequence of concurrent intracellular lipid accumulation and a deficiency in mitochondrial DHODH. DHODH, which is canonically embedded in the inner mitochondrial membrane to drive pyrimidine biosynthesis, was found to be redistributed to the cytosol in these cells. This cytosolic DHODH remained functionally active, exhibiting dihydroorotate catalytic activity and contributing to the pyrimidine biosynthesis pathway. Dihydroorotate (DHO) dihydroorotate dehydrogenase (DHODH).

Mitochondrial metabolic reprogramming, a core hallmark of CRC, has emerged as a key target for overcoming treatment resistance. Interventions targeting sugar, lipid, and nucleotide metabolic pathways can effectively disrupt the energy supply and biosynthetic capacity of tumor cells, providing new approaches to overcome the limitations of traditional treatments. At the glycometabolic level, the synergistic inhibition of glycolysis and oxidative phosphorylation has proven highly effective in countering the “Warburg effect” typical of CRC. Preclinical studies have shown that combining metformin (a mitochondrial complex I inhibitor) with 2-deoxyglucose (2-DG) can significantly inhibit the activation of the *p*-eIF2α/ATF4 signaling axis by blocking radiation-induced incomplete mitochondrial outer membrane permeabilization (MOMP), thereby reversing radio-resistance in tumor cells.^67^ The use of a liposome co-delivery system of metformin combined with 2-DG (M/D-Lipo) further enhances drug targeting, demonstrating synergistic effects in both locally advanced and metastatic CRC models, laying a solid foundation for clinical translation. In addition to targeting sugar metabolism, the regulation of lipid reprogramming is also crucial. Low molecular weight protein tyrosine phosphatase (LMW-PTP) is abnormally overexpressed in CRC, driving excessive lipid accumulation by positively regulating acetyl-CoA carboxylase and fatty acid synthase, thus promoting tumor invasion and metastasis. The natural compound violacein specifically binds to and inhibits LMW-PTP, inducing a metabolic phenotype shift in CRC cells from glycolysis to oxidative phosphorylation. This process is accompanied by increased mitochondrial oxygen consumption rate (OCR) and proton leak, ultimately disrupting the metabolic plasticity of tumor cells.^74^ However, these strategies face a core contradiction: the metabolic plasticity of tumor cells. Targeting a single pathway may trigger compensatory activation, leading to drug resistance. Developing combination therapies that can simultaneously inhibit multiple interconnected metabolic nodes is essential. Recent research also underscores the therapeutic potential of targeting nucleotide metabolism. In 5-fluorouracil (5-FU)-resistant CRC cells, mitochondrial-localized dihydroorotate dehydrogenase (DHODH) aberrantly translocates to the cytoplasm, resulting in two distinct effects: firstly, cytoplasmic DHODH catalyzes pyrimidine synthesis, forming a competitive antagonist against 5-FU; secondly, the loss of the mitochondrial lipid peroxidation defense mechanism significantly increases the cells' sensitivity to ferroptosis.^64^ Therefore, DHODH inhibitors represent a novel strategy to overcome chemotherapy resistance by synergistically inducing ferroptosis and reversing pyrimidine biosynthesis-mediated resistance. Breakthroughs in the development of novel delivery systems have further enhanced the targeting efficiency of metabolic intervention drugs. The oral colon-targeted nanoplatform M27-39 peptide conjugated with folic acid-functionalized mesoporous carbon nanoparticles (M27-39@FA-MCNs) utilizes folate receptor-mediated active targeting to deliver mitochondrial-toxic peptides specifically to CRC lesions. This system demonstrates efficient drug release in the acidic intestinal environment, significantly inhibiting tumor progression by disrupting mitochondrial energy metabolism and activating the p53/Caspase-3 apoptosis pathway.^71^ However, the clinical translation of such complex delivery systems faces a trade-off: while improving targeting, it is necessary to address the challenges of large-scale production and potential immunogenicity. This technological advancement addresses the off-target toxicity issues commonly associated with traditional chemotherapeutic drugs and provides a solid foundation for the clinical translation of metabolic interventions. Metabolic remodeling of the TME can also influence therapeutic efficacy. Recent evidence suggests that hypercholesterolemia induces CD8^+^ T cell exhaustion via the endoplasmic reticulum stress (ERS)-endoplasmic reticulum mitochondria contact-mitophagy axis. Enhanced ERS promotes the abnormal aggregation of proteins such as Fission 1 (Fis1)/B-cell receptor-associated protein 31 (Bap31) and Mitofusin 2 (MFN2)/Cytochrome *c* oxidase subunit 4 (COX4), leading to the overactivation of mitophagy. This, in turn, impairs ATP synthesis and induces metabolic disturbances that compromise T cell anti-tumor immunity.^75^ This innovatively links systemic metabolism and immunosuppression through organelle communication, providing a basis for combination therapy. These findings suggest that cholesterol-lowering therapy could restore immune surveillance within the TME, providing a theoretical basis for combining metabolic intervention with immunotherapy. The core limitation of current research lies in viewing metabolic pathways in isolation, while overcoming drug resistance requires us to consider them as a dynamic, adaptive network. Future therapeutic innovations must address three major challenges: metabolic plasticity, the complexity of the tumor microenvironment, and delivery efficiency. In summary, metabolic intervention strategies that simultaneously target three core components (including mitochondrial energy disorders [glucose metabolism], anabolic metabolism [lipid/nucleotide], and microenvironmental metabolism), offer a systematic approach to overcome the therapeutic bottlenecks in CRC. From a molecular perspective, targeting key nodes such as phosphorylated eukaryotic initiation factor 2 alpha (*p*-eIF2α)/activating transcription factor 4 (ATF4), LMW-PTP, and DHODH can effectively reverse treatment resistance. From a therapeutic perspective, immune metabolic regulation opens new dimensions for combination therapies. Collectively, these advances have accelerated the translation of metabolic-targeted therapies from basic research to clinical practice, offering promising new treatment options for patients with CRC (Figure 1).

### Apoptotic pathway activation

**Figure 2:**
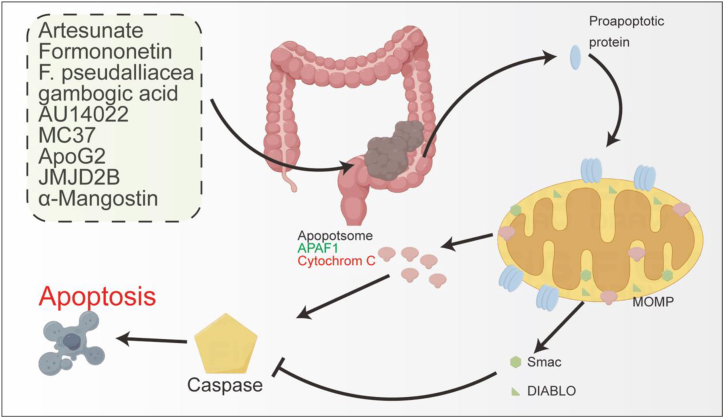
Dysregulation of the mitochondrial-mediated intrinsic apoptotic pathway plays a central role in CRC Various natural or synthetic compounds can regulate apoptosis signaling in CRC cells by influencing MOMP and thereby secreting factors such as Smac, DIABLO, APAF1, and Cytochrome C. second mitochondria-derived activator of caspases (Smac), direct IAP binding protein with low pI (DIABLO), mitochondrial outer membrane permeabilization (MOMP) (This figure is original by the author).

Dysregulation of the mitochondrial-mediated intrinsic apoptotic pathway plays a central role in CRC treatment resistance. At the core of this pathway is the dynamic balance within the BCL-2 protein family: the ratio between anti-apoptotic and pro-apoptotic members determines cell fate. Imbalances in this ratio hinder the activation of the MOMP pathway, contributing to apoptotic resistance. Targeting the mitochondrial-mediated apoptotic pathway has emerged as a core strategy in CRC treatment. This pathway overcomes apoptotic resistance in tumor cells through the restoration of Bcl-2 family balance, caspase cascade activation, and mitochondrial membrane permeability transition. Natural compounds have shown significant promise in modulating mitochondrial apoptosis. For instance, artesunate induces a concentration-dependent burst of ROS, reduces ΔΨm, promotes cytochrome *c* release, and activates the caspase-9/caspase-3 axis while upregulating the Bax/Bcl-2 ratio, ultimately triggering apoptosis in HCT-116 cells.^76^ Similarly, snake venom inhibits the anti-apoptotic function of Bcl-2 by phosphorylation, enhances second mitochondria-derived activator of caspases (Smac)/direct IAP binding protein with low pI (DIABLO) protein expression, and induces caspase-9/caspase-3 activation, promoting PARP cleavage and revealing its mitochondrial apoptosis mechanism.^77^ Formononetin is another natural compound that induces apoptosis through ROS-dependent mitochondrial depolarization while also inhibiting cancer cell migration and invasion.^78^^,^^79^ Laherradurin can disrupt mitochondrial structure and induce apoptosis in CRC cells.^80^ Additionally, the methanol extract of *Fusarium pseudalliacea* specifically induces apoptosis in HCT-116 cells by dose-dependently increasing the Bax/Bcl-2 ratio, activating caspase-3, and disrupting ΔΨm.^81^ Garcinia resin, containing gambogic acid, selectively activates the mitochondrial apoptosis pathway in CRC cells (SW480/Caco-2) by arresting the G2/M phase and elevating the Bax/Bcl-2 ratio, with minimal toxicity to normal colon cells (CCD841 CoN).^82^ While many natural compounds have shown the potential to induce apoptosis in preclinical models, a key paradox is that their multi-target nature is both an advantage and a major obstacle to clinical translation. This broad-spectrum action, while helpful in overcoming drug resistance, makes it difficult to elucidate their primary mechanism of action and poses challenges to accurate efficacy assessment and side effect management. Synthetic small molecule compounds also present unique advantages by precisely targeting key apoptosis regulatory nodes. The novel benzoxazole derivative AU14022 enhances p53 Ser15 phosphorylation, promotes the expression of p21 and BAX, induces mitochondrial dysfunction, and facilitates cytochrome *c* release. When combined with radiotherapy, it synergistically induces G2/M arrest, significantly enhancing the radiosensitivity of HCT-116 xenografts.^83^ The monocarbonyl curcumin analog MC37 acts through multiple mechanisms: it inhibits microtubule assembly, downregulates CDK1 to arrest cells in the G2/M phase, and activates the caspase-9/3 cascade by increasing the Bax/Bcl-2 ratio, thus achieving the dual regulation of apoptosis and cell-cycle arrest.^84^ Among novel strategies targeting the Bcl-2 family, the gossypol derivative ApoG2 selectively blocks Mcl-1-Bax interactions, thereby promoting mitochondrial cytochrome *c* release and activating caspase-3/7. This apoptosis-inducing ability has been demonstrated in HT29, SW480, and HCT116 cells.^85^ Intervention in key apoptosis-regulating proteins offers new avenues for precision medicine. Silencing JMJD2B activates apoptosis through two mechanisms: by downregulating Bcl-2 to trigger the mitochondrial pathway; and via caspase-8-mediated Bid cleavage to engage the death receptor pathway. This ERS-independent dual mechanism offers a promising strategy to overcome drug resistance.^86^ Research on the cancer stem cell marker leucine-rich repeat-containing g-protein coupled receptor 5 (LGR5) has shown that its deletion inhibits β-catenin nuclear translocation and downregulates c-myc/cyclin D expression, leading to ΔΨm collapse and apoptosis. Treatment with Wnt3a reverses LGR5-depletion-induced apoptosis, underscoring the central role of Wnt/β-catenin signaling in LGR5-regulated mitochondrial apoptosis.^87^ These treatment strategies demonstrate a trend toward shifting from broad-spectrum cytotoxic drugs to precise intervention at specific apoptosis points. However, how intratumoral heterogeneity affects the efficacy of these precision strategies remains unclear. Innovative delivery systems and combination strategies are also advancing apoptosis induction. A novel marine-derived peptide, P6, induces cell-cycle arrest and apoptosis by activating the p38-MAPK pathway, with confirmed anti-tumor activity in CRC xenograft models, providing a new paradigm for peptide-based drug therapy.^88^ Mitochondrial-targeted delivery of curcumin simultaneously modulates multiple pathways, including inhibiting survivin expression, increasing p53 accumulation, elevating the Bax/Bcl-2 ratio, and activating caspase-9, resulting in highly effective killing of human CRC LoVo cells.^89^ In combination therapy, the 7-deoxypanclasistatin analog JCTH-4 induces mitophagy and ROS burst, leading to cytochrome *c* release and ΔΨm collapse. When combined with tamoxifen, JCTH-4 synergistically enhances autophagy and apoptosis, demonstrating efficacy in both wild-type (HCT116) and mutant (HT-29) p53 cells, with minimal toxicity to normal colonic fibroblasts (CCD-18Co).^90^ Even more promising is α-mangostin, which induces apoptosis in DLD-1 cells through a caspase-independent pathway by promoting endonuclease-G release, inhibiting protein kinase B (Akt) phosphorylation, and upregulating miR-143 (targeting Erk5). When combined with 5-FU (2.5 μM), it significantly enhances growth inhibition, demonstrating potential for chemosensitivity.^91^ These advances collectively support the transformation of mitochondrial apoptosis-targeting therapies from basic research to clinical practice, providing new opportunities to improve the prognosis of patients with CRC (Figure 2).

### Regulation of mitochondrial dynamics

**Figure 3:**
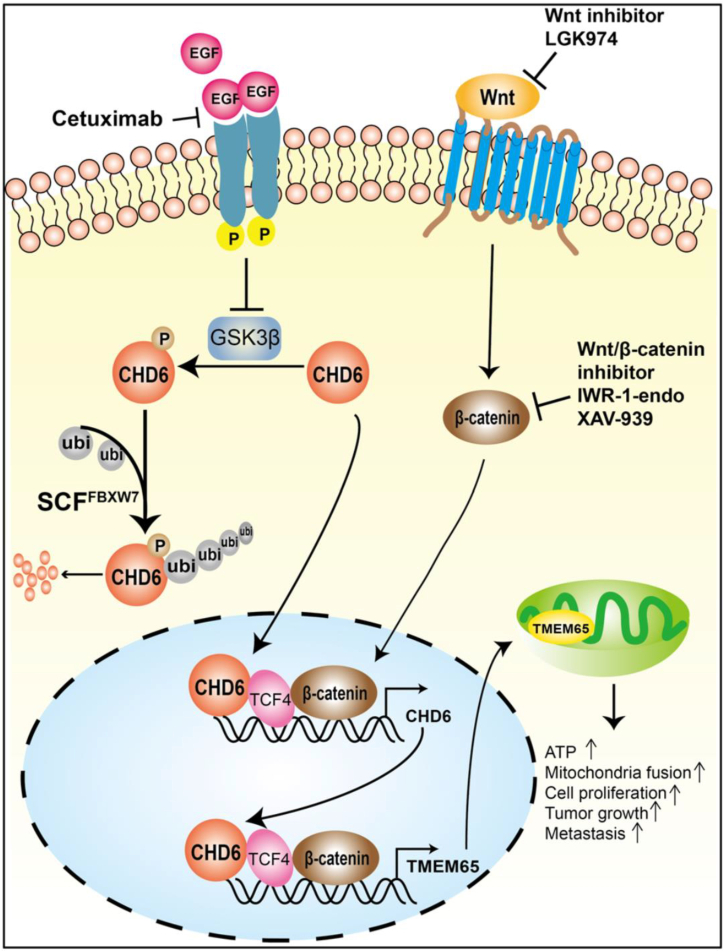
CHD6-TMEM65 axis is regulated by both EGF and Wnt signaling pathways in promoting cancer growth Reproduced with permission from ref.^93^ CHD6 is highly expressed in CRC. Moreover, EGF signaling was found to enhance CHD6 stability by suppressing its ubiquitin-mediated degradation. This stabilization occurs through a specific mechanism: EGF signaling inhibits GSK3beta activity, which prevents the formation of a phosphodegron on CHD6. Consequently, the recognition and ubiquitination of CHD6 by the E3 ligase FBXW7 is hindered, thereby blocking its degradation. Functionally, CHD6, as a chromatin remodeler, binds to the Wnt signaling transcription factor TCF4 to promote the transcription of TMEM65. The CHD6-TMEM65 axis is often dysregulated in cancer. Importantly, the combined administration of the Wnt inhibitor LGK974 and the anti-EGFR antibody cetuximab significantly suppressed the growth of patient-derived CRC xenografts, highlighting a potential therapeutic strategy.

Precise regulation of mitochondrial dynamics, particularly the balance between fusion and fission, is crucial for maintaining cellular energy homeostasis. Dysregulation of these processes in CRC has emerged as a novel therapeutic target. TRAP1 is negatively correlated with mitochondrial-encoded respiratory chain proteins at both the transcriptional and translational levels. Knockdown of TRAP1 can enhance mitochondrial biosynthesis through the PGC-1α/TFAM signaling pathway, thereby promoting the formation of new functional mitochondria.^92^ Recent studies have demonstrated that lycorine, by targeting the C-terminal domain of isocitrate dehydrogenase 1 (IDH1), disrupts its interaction with the deacetylase sirtuin 1 (SIRT1), leading to the significant promotion of IDH1 acetylation. This modification triggers mitochondrial oxidative stress, causing damage to the mitochondrial membrane and enhancing mitochondrial fission. Specific knockdown of either IDH1 or SIRT1 alleviates lycorine-induced mitochondrial fragmentation. Moreover, combining lycorine with the SIRT1 inhibitor nicotinamide (NAM) synergistically enhances apoptosis in CRC cells, confirming that the IDH1-SIRT1 axis is a key regulatory hub in mitochondrial dynamics.^47^ Epigenetic regulation also plays a pivotal role in mitochondrial structure. The chromatin remodeling factor chromodomain helicase DNA-binding protein 6 (CHD6) is abnormally overexpressed in CRC, and its stability is regulated through the EGF-GSK3β-FBXW7 signaling axis. EGF inhibits GSK3beta activity, preventing CHD6 phosphorylation and degradation, which maintains CHD6 protein levels by escaping FBXW7-mediated ubiquitination. As a transcriptional coactivator, CHD6 binds to TCF4, a key factor in the Wnt pathway, to directly promote the expression of TMEM65, a mitochondrial inner membrane protein. TMEM65 acts as a hub in energy metabolism, promoting tumor cell survival by regulating ATP synthesis and mitochondrial fusion. Wnt ligands can feedback-activate CHD6 transcription, establishing a CHD6-TMEM65 positive feedback loop. A dual-targeted intervention using the Wnt inhibitor LGK974 in combination with the anti-EGFR antibody cetuximab synergistically blocks this pathway, significantly inhibiting the growth of CRC patient-derived xenografts^93^ (Figure 3). Kinetic interventions targeting specific mutations show clinical promise. In BRAF V600E-mutant CRC (affecting 8–12% of patients), the compound HAMLET enhances FOLFOX sensitivity by reprogramming mitochondrial respiration. Notably, HAMLET potently inhibits OXPHOS in BRAF wild-type cells but has attenuated effects in BRAF mutant cells such as WiDr. Consequently, the HAMLET/FOLFOX combination synergistically reduces viability, with greater efficacy in BRAF wild-type models. This differential response, dictated by intrinsic mitochondrial metabolic phenotypes, highlights their potential as biomarkers for personalizing combination therapy.^94^ Aberrant expression of ZNF746 (PARIS) drives CRC progression by disrupting mitochondrial fusion-fission balance, primarily through suppressing MFN1, MFN2, and PGC1α. Clinically, ZNF746 is overexpressed in tumors, and its silencing restores mitochondrial homeostasis to increase 5-FU sensitivity. Notably, melatonin synergizes with 5-FU by inhibiting ZNF746 signaling, collectively revealing a novel strategy to overcome chemoresistance.^65^

In summary, strategies for regulating mitochondrial dynamics in CRC focus on key molecular nodes: IDH1 acetylation (a driver of fission), the chromodomain helicase DNA-binding protein 6 (CHD6)-transmembrane protein 65 (TMEM65) axis (fusion maintenance), zinc finger protein 746 (ZNF746, fusion inhibition), and human α-lactalbumin Made Lethal to Tumor cells (HAMLET, respiratory reprogramming).

### Mitophagy

Mitophagy, a key quality control mechanism for selectively removing damaged mitochondria, plays a dual role in the pathogenesis, drug resistance, and immune evasion of CRC. Precise regulation of mitophagy has emerged as a potential strategy to overcome therapeutic challenges. The PINK1/Parkin pathway is a central hub in this process: upon loss of ΔΨm, PINK1 accumulates on the outer mitochondrial membrane, recruiting and activating the E3 ubiquitin ligase Parkin. Parkin subsequently ubiquitinates mitochondrial proteins, promoting the formation of autophagosomes. Clinical studies have shown that Parkin expression is significantly reduced in CRC tissues and correlates with poor prognosis.^51^^,^^95^ Mechanistically, the deubiquitinating enzyme USP26 enhances CRC tumorigenesis by binding to the K129 site of Parkin, inhibiting its activation and blocking mitophagic flux.^51^ In contrast, the piRNA piR-823 binds to PINK1, promoting its ubiquitination and degradation, thereby weakening the PINK1/Parkin pathway and resulting in the accumulation of damaged mitochondria.^95^ Additionally, the G protein-coupled receptor 176 (GPR176)- GNAS complex locus complex (GNAS) complex inhibits BNIP3L expression through cAMP/PKA signaling, further preventing Parkin’s mitochondrial translocation, and establishing a three-tiered “receptor-signal-effector” regulatory network.^50^ These findings collectively highlight the central role of negative regulation of mitophagy in CRC progression.

Mitophagy has been shown to promote survival in cancer stem cells. CD133^+^/CD44^+^ CRC stem cells overexpress Bcl-2/adenovirus E1B 19 kDa-interacting protein 3-like (BNIP3L), which clears doxorubicin-induced damaged mitochondria through enhanced selective autophagy, thereby maintaining low mitochondrial superoxide levels and significantly reducing chemotherapy sensitivity.^96^ Similarly, the AMP-activated protein kinase (AMPK)-S-phase kinase-associated protein 2 (Skp2) axis protects CRC cells from mitochondrial apoptosis by activating Parkin and enhancing autophagic flux. This “autophagy barrier” effect is particularly pronounced in the MSI-H subtype: the deubiquitinating enzyme ubiquitin-specific peptidase 14 (USP14) stabilizes the Bcl-2–associated anthogene gene 4 (BAG4) protein via K48 deubiquitination, preventing Parkin recruitment to damaged mitochondria and contributing to oxaliplatin resistance.^97^ Targeting these mechanisms, δ-valerate betaine and oxymatrine induce mitophagy-dependent apoptosis by activating the PINK1/Parkin pathway, offering a promising strategy to overcome drug resistance.^52^^,^^98^

Mitophagy-related molecular markers have demonstrated significant value in prognostic prediction and treatment stratification in CRC. A risk model based on 10 mitophagy genes (e.g., activating molecule in beclin-1-regulated autophagy [AMBRA1], autophagy-related 14 [ATG14], microtubule-associated protein 1 light chain 3 beta [MAP1LC3B]) can effectively stratify patients with CRC into high-risk and low-risk groups. The high-risk group is associated with significantly shortened overall survival and increased sensitivity to targeted therapies such as bosutinib and sunitinib, whereas the low-risk group is more likely to benefit from immunotherapy.^99^ Among these markers, C-X-C motif chemokine ligand 3 (CXCL3) has been identified as a novel oncogenic factor. Its overexpression promotes ATG14-dependent mitophagy via the activation of the p38-MAPK pathway, which enhances CRC cell proliferation, migration, and chemotherapy resistance.^100^ Moreover, the gene combination ubiquitin C-terminal hydrolase L1 (UCHL1)/heart shock protein A1A (HSPA1A)/plectin (PLEC) shows a diagnostic area under the curve (AUC) > 0.9, highlighting its potential for clinical application.^101^ Single-cell sequencing further revealed that mitophagy-related pathways are commonly activated in cancer cells, delineating the C2 (high autophagy) subtype, which is characterized by distinct metabolic features and an immunologically “cold” phenotype.^102^ These findings are advancing the development of personalized treatment strategies. For example, statins exert anticancer effects by inducing mitochondrial dysfunction; however, cancer cells can circumvent this effect by activating PINK1/Parkin-mediated autophagy. Combining statins with autophagy inhibitors has been shown to significantly enhance therapeutic efficacy.^103^

**Figure 4:**
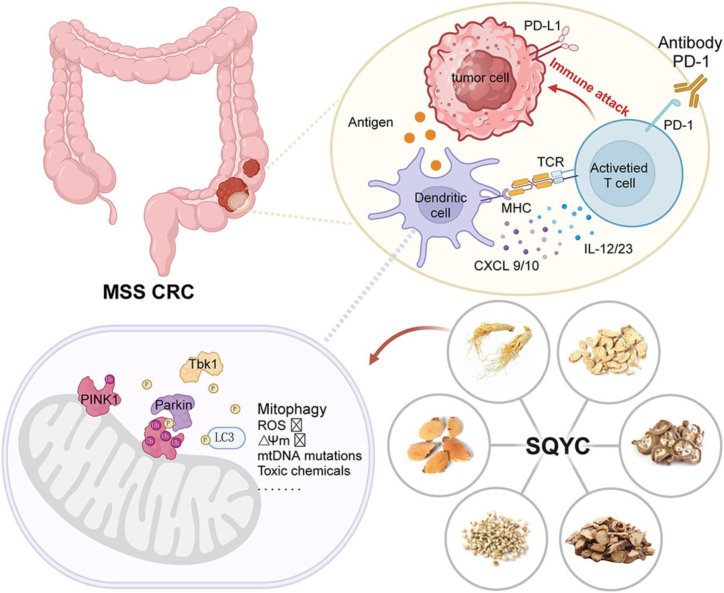
Traditional Chinese medicine combined with tumor immunology mediates mitophagy in CRC SQYC formula improves the efficacy of PD-1 monoclonal antibodies in CRC by regulating dendritic cell mitophagy via the PINK1-Parkin pathway. Reproduced with permission from ref.^105^

Natural compounds and traditional Chinese medicine (TCM) formulas demonstrate significant therapeutic potential by regulating mitophagy through multiple molecular targets. The TCM compound Tong-Xie-Yao-Fang (TXYF) induces mitophagy by activating the PINK1/Parkin pathway, reverses epithelial-mesenchymal transition, and significantly inhibits colitis-associated carcinogenesis (*p* < 0.01).^104^ Similarly, Shenqi Yichang Fang (SQYC) promotes PINK1-Parkin-dependent mitophagy in dendritic cells, enhances mitochondrial energy metabolism, and improves tumor-infiltrating lymphocyte function, thereby increasing the sensitivity of microsatellite stable (MSS)-type CRC to programmed cell death protein 1 (PD-1) inhibitors.^105^ Among individual compounds, oxymatrine inhibits CRC liver metastasis via dual mechanisms: by suppressing the leucine-rich pentatricopeptide repeat containing (LRPPRC) protein, promoting Parkin mitochondrial translocation, and inhibiting the NLRP3 inflammasome. Carnitine, on the other hand, synergistically promotes mitophagy-dependent apoptosis by activating the AMPK-SIRT4 axis.^106^ In contrast, strigolactone analogs specifically inhibit mitophagy by blocking the fusion of autophagosomes with lysosomes.^107^ In summary, the regulation of mitophagy in CRC therapy presents a “double-edged sword” characteristic. On the one hand, targeting USP26-Parkin (inhibitor), BNIP3L (siRNA), or SQYC (immunopotentiation) can restore its tumor-suppressive function. On the other hand, inhibiting the CXCL3-ATG14 axis or combining statins with autophagy inhibitors can mitigate the pro-survival effects of mitophagy (Figure 4).

### Targeting mitochondrial transfer (MT)

MT is an emerging mechanism that plays an important role in health and disease.^108^^,^^109^ MT regulates tumor metabolism and plays a critical role in chemotherapy resistance and stemness maintenance in CRC. A pioneering study by Sasaki et al.^110^ demonstrated that bone marrow mesenchymal stem cells (BM-MSCs) transfer functional mitochondria to 5-FU-damaged CRC cells via an oxidized high-mobility group box 1 (HMGB1)-dependent pathway. This process is driven by the HMGB1/NF-κB signaling axis. Inhibition of MT using anti-HMGB1 antibodies significantly reduces MT, while NF-κB inhibitors downregulate MT-related gene expression. CRC cells that received mitochondria not only restored mitochondrial function but also acquired enhanced stemness characteristics (e.g., CD44^+^ and CD133+) and resistance to 5-FU. Furthermore, in a subcutaneous xenograft tumor model, MT in host BM-MSCs diminished CRC chemotherapy sensitivity. These findings provide direct evidence that targeting MT could overcome CRC drug resistance. While direct studies of MT in CRC remain limited, research in other cancer types has revealed shared mechanisms and therapeutic potential. For example, in ovarian cancer, cancer-associated mesenchymal stem cells (CA-MSCs) induce tumor heterogeneity and promote metastasis through MT,^111^ where mitochondrial donation rescues the phenotype of “mito-poor” cancer cells. This rescue restores their proliferative capacity, chemotherapy resistance, and cellular respiration. These changes promote broader tumor growth.^111^ In breast cancer (BC), cancer-associated fibroblasts (CAFs) transport mitochondria through tunneling nanotubes (TNTs), enhancing cancer cell oxidative phosphorylation (OXPHOS) and migration,^112^ with recent evidence showing that mitochondrial transfer from adipose stem cells can drive multi-drug resistance by increasing OXPHOS and ABC transporter expression.^113^ Combining drugs that inhibit TNTs with immune checkpoint inhibitors can significantly improve the treatment outcomes of breast cancer.^109^ New research has found that nerve cells can increase the metastatic ability of tumors by transferring mitochondria to tumor cells.^114^ A pivotal mechanism of immune evasion involves mitochondrial transfer from cancer cells to tumor-infiltrating lymphocytes, which impairs T cell function and promotes senescence, revealing a novel role for MT in modulating the immune TME.^113^ The crosstalk between stromal and cancer cells via MT is facilitated by intercellular structures such as TNTs and extracellular vesicles, with gastric cancer studies highlighting that extracellular matrix stiffness can promote MSC-mediated mitochondrial transfer to confer chemotherapy resistance,^115^ leading to significant metabolic reprogramming in recipient cells. MT shows distinct regulatory patterns across different tumors. In CRC, the oxidative state of HMGB1 is a specific trigger for MT, whereas in prostate cancer, MT is induced by the lactate shuttle,^116^ and in BC, MT relies on the physical structure of TNTs.^117^ Additionally, mitochondrial transfer between hepatocellular carcinoma cells, promoted by HMGB1 under hypoxia via RHOT1 and RAC1, enhances migration and invasion,^118^ underscoring the role of the hypoxic TME. These cross-cancer differences suggest that developing organ-specific strategies will be crucial for targeting MT in CRC. Current CRC treatment strategies targeting metastasis focus on three main approaches: (1) Blocking inductive signaling: Monoclonal antibodies or small molecule inhibitors targeting oxidized HMGB1 (e.g., glycyrrhizic acid analogs) can disrupt the crosstalk between BM-MSCs and CRC cells, with preclinical models demonstrating the restoration of 5-FU sensitivity. (2) Inhibiting metastatic pathways: Actin polymerization inhibitors, such as cytochalasin D, can block TNT formation (based on BC models) and hinder mitochondrial trafficking to CRC cells. (3) Intervention in metabolic reprogramming: Following metastasis, CRC cells become reliant on OXPHOS, and combining complex I inhibitors with 5-FU shows potential for synergistic tumor killing. However, significant challenges remain in the field include: (1) the diversity of donor cells for MT within the CRC microenvironment (with the roles of CAFs and endothelial cells yet to be fully elucidated); (2) the unclear epigenetic mechanisms that drive MT-induced stemness; and (3) the lack of *in vivo* real-time tracking technologies. Thus, future research will need to integrate CRC organoid models with single-cell metabolomics to better understand the spatiotemporal dynamics of MT. Furthermore, efforts should focus on exploring drugs targeting HMGB1 oxidoreductase to disrupt MT-inducing signals at their source. Mitochondrial trafficking serves as a key hub for metabolic interactions within the CRC microenvironment, directly influencing stemness and drug resistance. Although research targeting MT in CRC is still in its early stages, evidence from other cancers suggests that targeting MT holds broad translational potential. In the future, mechanistic innovations such as the development of mitochondrial “decoy” nanoparticles and clinical validation efforts, including the identification of MT biomarkers, will be essential to advance the concept of “blocking mitochondrial theft” from its conceptualization to clinical application in CRC (Table 1).

**Table 1:** Summary of studies on mitochondrial transfer in tumors

Cancer type	Donor cells	Transfer mechanism	Core features	Key targets	Reference
Colorectal cancer	Bone marrow mesenchymal stem cells	Exosome and TNTs	Enhanced stemness characteristics and chemotherapy resistance	Oxidized HMGB1	Sasaki et al.^110^
Ovarian cancer	Cancer-associated mesenchymal stromal cells	TNTs	Driving tumor heterogeneity and metastasis	ANGPTL3	Frisbie et al.^111^
Breast cancer	Cancer-associated fibroblasts	TNTs	Increased OXPHOS and promote 3D migration	Mitochondrial complex I	Goliwas et al.^112^
Breast cancer	Normal mammary epithelial cells	TNTs	Suppressed cancer cell proliferation and reversal of malignant phenotypes	Undefined	Bjerring et al.^117^
Prostate cancer	Cancer-associated fibroblasts	TNTs	Induction of mitochondrial hyperfunction and ROS accumulation	Lactate transporter	Ippolito et al.^116^

TNTs, tunneling nanotubes; OXPHOS, oxidative phosphorylation; HMGB1, high-mobility group box 1; ANGPTL3, angiopoietin-like protein 3; ROS, reactive oxygen species.

## Clinical translational progress

The advancement of mitochondrial research in clinical translational oncology is progressively shifting from basic mechanistic insights to practical applications. Liquid biopsy technologies, in particular, have shown substantial promise in CRC. Studies indicate that the combined analysis of nuclear DNA and mitochondrial-derived cell-free DNA (cfDNA) can effectively monitor treatment response and tumor recurrence. For example, reductions in cfDNA levels and alterations in fragment length following chemotherapy are positively correlated with therapeutic efficacy.^119^ Plasma copy numbers of mitochondrial cfDNA are higher in healthy individuals than in patients with CRC, highlighting a significant inverse correlation that provides valuable insights for non-invasive diagnostics. Furthermore, mtDNA content in leukocytes has been identified as an independent prognostic marker. High mtDNA content is significantly associated with reduced overall survival and increased recurrence risk in patients with CRC, and correlates with an immunosuppressive microenvironment. This marker not only complements tumor-node-metastasis (TNM) staging for prognostic prediction but also serves as a predictor of the likelihood of benefit from adjuvant chemotherapy.^120^ In BC, exercise interventions significantly increase levels of the mitochondrial-derived peptide mitochondrial open reading frame of the 12S rRNA-c (MOTS-c) in non-Hispanic white survivors, improving both body composition and metabolic markers, although these effects vary by ethnicity.^121^ Regarding treatment-related toxicities, studies have found that chemoradiotherapy can impair muscle mitochondrial function (manifested by decreased creatine phosphate recovery) and reduce systemic fitness.^122^ However, exercise training has been shown to maintain skeletal muscle mitochondrial content and capillary density in patients with BC, alleviating chemotherapy-induced fatigue.^123^ In PCa therapy, fatigue induced by androgen deprivation combined with radiotherapy is linked to decreased adenosine triphosphate (ATP) coupling efficiency in peripheral blood mononuclear cells and dysregulated brain metabolic homeostasis, suggesting coordinated impairment of both central and peripheral mitochondrial function.^124^ Although early clinical trials of AMPK activator ASP4132, an inhibitor of mitochondrial complex I, were limited by dose-limiting toxicities, they nonetheless demonstrated the feasibility of targeted intervention.^125^ Translationally, nutritional intervention strategies have shown progress. Lipid replacement therapy combined with antioxidants significantly alleviates chemotherapy-related fatigue by preserving mitochondrial membrane integrity.^126^ Furthermore, prospective studies have shown that an elevated leukocyte mtDNA copy number prior to PCa diagnosis is associated with an increased risk of early, non-invasive tumors and elevated prostate-specific antigen levels, potentially reflecting early disturbances in the tissue microenvironment.^127^ Collectively, these findings underscore the transformative potential of mitochondrial-targeted strategies in cancer. They pave the way for the development of personalized diagnostic tools, toxicity management approaches, and novel therapeutic targets, offering a new dimension for precision medicine in oncology (Table 2).

**Table 2:** Summary of clinical studies related to mitochondria and cancer

Research topics	Cancer types	Research design	Clinical significance	Reference
First-in-human trial of mitochondrial complex I inhibitor (ASP4132)	Advanced solid tumors	RCT	Targeting mitochondrial metabolism has been demonstrated as feasible, but severe toxicity limits dose escalation, suggesting a potentially narrow therapeutic window for such drugs.	Janku et al.^125^
Mitochondrial DNA Copy Number and Prostate Cancer Risk	PC	OCS	mtDNA copy number may reflect early damage to prostate gland structure, rather than late-stage tumor burden, or serve as a biomarker for early disease.	Moore et al.^127^
Analysis of Nuclear and Mitochondrial DNA in Tissue and Liquid Biopsies	CRC	OCS	Liquid biopsy (especially nuclear cfDNA) is an effective complement or alternative to tissue biopsy. Changes in mtDNA copy number in cancer warrant further investigation.	Haupts et al.^119^
Cancer-Related Fatigue and Mitochondrial Dysfunction	PC	OCS	For the first time, treatment-related fatigue has been demonstrated in clinical and animal models to be associated with central and peripheral mitochondrial dysfunction, providing a target for intervention.	Feng et al.^124^
Protective Effects of Exercise Training on Muscle Mitochondria During Chemotherapy	BC	RCT	Supervised exercise during chemotherapy is feasible and can mitigate the toxic side effects of treatment on muscle mass and function, improving patients' quality of life.	Mijwel et al.^123^
Effects of Exercise on the Mitochondrial-Derived Peptide MOTS-c	BC	RCT	The effects of exercise on mitochondrial function may vary racially, highlighting the importance of personalized exercise prescriptions and biomarker research.	Dieli-Conwright et al.^121^
Effects of Neoadjuvant Chemotherapy on Performance and Muscle Mitochondria	CRC	OCS	Chemotherapy and radiotherapy directly impair muscle mitochondrial function, which may be a significant cause of decreased physical performance and increased fatigue in patients, suggesting the need for pre-rehabilitation interventions.	West et al.^122^

Randomized controlled trial (RCT), observational cohort study (OCS), prostate cancer (PC), colorectal cancer (CRC), and breast cancer (BC).

## Conclusion

Targeted mitochondrial therapy is an emerging paradigm for overcoming drug resistance in CRC. Future clinical translation faces three major challenges: tumor heterogeneity, metabolic symbiosis within the tumor microenvironment, and targeted delivery efficiency. Key directions for addressing these challenges include: developing combination therapies targeting multiple mitochondrial nodes, integrating them with immunotherapy to reshape the immune microenvironment, and leveraging organoid models and nanotechnology to advance personalized treatment. By focusing on these areas, mitochondrial-targeted strategies hold the promise of groundbreaking advancements in CRC treatment.

## Acknowledgments

No funding has been received for this study.

## Author contributions

Hao Che and Xiao-Jiang Ying designed this study. Hao Che, Zhen-Jun Li, Ling Xu, Yangbing Du, SongOu Zhang, and Xiao-Jiang Ying prepared this article. All authors reviewed the article.

## Declaration of interests

The authors declare no competing interests.

## References

[1] Arnold M., Sierra M.S., Laversanne M., Soerjomataram I., Jemal A., Bray F. (2017). Global patterns and trends in colorectal cancer incidence and mortality. Gut.

[2] Li N., Lu B., Luo C., Cai J., Lu M., Zhang Y., Chen H., Dai M. (2021). Incidence, mortality, survival, risk factor and screening of colorectal cancer: A comparison among China, Europe, and northern America. Cancer Lett..

[3] Mauri G., Sartore-Bianchi A., Russo A.G., Marsoni S., Bardelli A., Siena S. (2019). Early-onset colorectal cancer in young individuals. Mol. Oncol..

[4] Siegel R.L., Jakubowski C.D., Fedewa S.A., Davis A., Azad N.S. (2020). Colorectal Cancer in the Young: Epidemiology, Prevention, Management. Am. Soc. Clin. Oncol. Educ. Book..

[5] Leowattana W., Leowattana P., Leowattana T. (2023). Systemic treatment for metastatic colorectal cancer. World J. Gastroenterol..

[6] Paty P.B., Garcia-Aguilar J. (2022). Colorectal cancer. J. Surg. Oncol..

[7] Shin A.E., Giancotti F.G., Rustgi A.K. (2023). Metastatic colorectal cancer: mechanisms and emerging therapeutics. Trends Pharmacol. Sci..

[8] Biller L.H., Schrag D. (2021). Diagnosis and Treatment of Metastatic Colorectal Cancer: A Review. JAMA.

[9] Salva de Torres C., Baraibar I., Saoudi González N. (2024). Current and Emerging Treatment Paradigms in Colorectal Cancer: Integrating Hallmarks of Cancer. Int. J. Mol. Sci..

[10] Fan A., Wang B., Wang X., Nie Y., Fan D., Zhao X., Lu Y. (2021). Immunotherapy in colorectal cancer: current achievements and future perspective. Int. J. Biol. Sci..

[11] Senousy M.A., Shaker O.G., Ayeldeen G., Radwan A.F. (2024). Association of lncRNA MEG3 rs941576 polymorphism, expression profile, and its related targets with the risk of obesity-related colorectal cancer: potential clinical insights. Sci. Rep..

[12] Nikmanesh F., Sarhadi S., Dadashpour M., Asghari Y., Zarghami N. (2020). Omics Integration Analysis Unravel the Landscape of Driving Mechanisms of Colorectal Cancer. Asian Pac. J. Cancer Prev..

[13] Lotfi-Attari J., Pilehvar-Soltanahmadi Y., Dadashpour M., Alipour S., Farajzadeh R., Javidfar S., Zarghami N. (2017). Co-Delivery of Curcumin and Chrysin by Polymeric Nanoparticles Inhibit Synergistically Growth and hTERT Gene Expression in Human Colorectal Cancer Cells. Nutr. Cancer.

[14] Sharifi-Azad M., Fathi M., Cho W.C., Barzegari A., Dadashi H., Dadashpour M., Jahanban-Esfahlan R. (2022). Recent advances in targeted drug delivery systems for resistant colorectal cancer. Cancer Cell Int..

[15] Firouzi Amandi A., Jokar E., Eslami M., Dadashpour M., Rezaie M., Yazdani Y., Nejati B. (2023). Enhanced anti-cancer effect of artemisinin- and curcumin-loaded niosomal nanoparticles against human colon cancer cells. Med. Oncol..

[16] Johdi N.A., Sukor N.F. (2020). Colorectal Cancer Immunotherapy: Options and Strategies. Front. Immunol..

[17] Margalit O., Stemmer A., Chapin W.J., Shacham-Shmueli E., Kopetz S., Andre T., Overman M.J., Pietrantonio F., Boursi B. (2024). Duration of immunotherapy in dMMR/MSI-H metastatic colorectal cancer patients. Eur. J. Cancer.

[18] Oliveira A.F., Bretes L., Furtado I. (2019). Review of PD-1/PD-L1 Inhibitors in Metastatic dMMR/MSI-H Colorectal Cancer. Front. Oncol..

[19] Amonkar M.M., Chase M., Myer N.M., Wang T., Turzhitsky V., Spira A. (2023). Real-world treatment patterns and clinical outcomes for chemotherapy-based regimens in first-line MSI-H/dMMR metastatic colorectal cancer. Cancer Treat. Res. Commun..

[20] Chen J., Duan S., Wang Y., Ling Y., Hou X., Zhang S., Liu X., Long X., Lan J., Zhou M. (2024). MYG1 drives glycolysis and colorectal cancer development through nuclear-mitochondrial collaboration. Nat. Commun..

[21] Zhao Y., Guo X., Zhang L., Wang D., Li Y. (2024). Mitochondria: a crucial factor in the progression and drug resistance of colorectal cancer. Front. Immunol..

[22] San-Millán I., Brooks G.A. (2017). Reexamining cancer metabolism: lactate production for carcinogenesis could be the purpose and explanation of the Warburg Effect. Carcinogenesis.

[23] Zhang W., Xia M., Li J., Liu G., Sun Y., Chen X., Zhong J. (2025). Warburg effect and lactylation in cancer: mechanisms for chemoresistance. Mol. Med..

[24] Fendt S.M. (2024). 100 years of the Warburg effect: A cancer metabolism endeavor. Cell.

[25] Zhong X., He X., Wang Y., Hu Z., Huang H., Zhao S., Wei P., Li D. (2022). Warburg effect in colorectal cancer: the emerging roles in tumor microenvironment and therapeutic implications. J. Hematol. Oncol..

[26] Nicolini A., Ferrari P. (2024). Involvement of tumor immune microenvironment metabolic reprogramming in colorectal cancer progression, immune escape, and response to immunotherapy. Front. Immunol..

[27] Seyfried T.N., Lee D.C., Duraj T., Ta N.L., Mukherjee P., Kiebish M., Arismendi-Morillo G., Chinopoulos C. (2025). The Warburg hypothesis and the emergence of the mitochondrial metabolic theory of cancer. J. Bioenerg. Biomembr..

[28] Sun Y., Wang H., Cui Z., Yu T., Song Y., Gao H., Tang R., Wang X., Li B., Li W., Wang Z. (2025). Lactylation in cancer progression and drug resistance. Drug Resist. Updat..

[29] Zhang J., Ouyang F., Gao A., Zeng T., Li M., Li H., Zhou W., Gao Q., Tang X., Zhang Q. (2024). ESM1 enhances fatty acid synthesis and vascular mimicry in ovarian cancer by utilizing the PKM2-dependent warburg effect within the hypoxic tumor microenvironment. Mol. Cancer.

[30] Jing Z., Liu Q., He X., Jia Z., Xu Z., Yang B., Liu P. (2022). NCAPD3 enhances Warburg effect through c-myc and E2F1 and promotes the occurrence and progression of colorectal cancer. J. Exp. Clin. Cancer Res..

[31] Yang W., Zheng Y., Xia Y., Ji H., Chen X., Guo F., Lyssiotis C.A., Aldape K., Cantley L.C., Lu Z. (2012). ERK1/2-dependent phosphorylation and nuclear translocation of PKM2 promotes the Warburg effect. Nat. Cell Biol..

[32] Wang Q., Guo X., Li L., Gao Z., Su X., Ji M., Liu J. (2020). N(6)-methyladenosine METTL3 promotes cervical cancer tumorigenesis and Warburg effect through YTHDF1/HK2 modification. Cell Death Dis..

[33] Lis P., Dyląg M., Niedźwiecka K. (2016). The HK2 Dependent “Warburg Effect” and Mitochondrial Oxidative Phosphorylation in Cancer: Targets for Effective Therapy with 3-Bromopyruvate. Molecules.

[34] Zhang K., Chen Y., Huang X., Qu P., Pan Q., Lü L., Jiang S., Ren T., Su H. (2016). Expression and clinical significance of cytochrome c oxidase subunit IV in colorectal cancer patients. Arch. Med. Sci..

[35] Ionescu V.A., Gheorghe G., Bacalbasa N., Chiotoroiu A.L., Diaconu C. (2023). Colorectal Cancer: From Risk Factors to Oncogenesis. Medicina (Kaunas).

[36] Feng W.Q., Zhang Y.C., Xu Z.Q., Yu S.Y., Huo J.T., Tuersun A., Zheng M.H., Zhao J.K., Zong Y.P., Lu A.G. (2023). IL-17A-mediated mitochondrial dysfunction induces pyroptosis in colorectal cancer cells and promotes CD8 + T-cell tumour infiltration. J. Transl. Med..

[37] Amin T., Sharma R.P., Mir K.B., Slathia N., Chhabra S., Tsering D., Kotwal P., Bhagat M., Nandi U., Parkesh R. (2023). Quinoxalinone substituted pyrrolizine (4h)-induced dual inhibition of AKT and ERK instigates apoptosis in breast and colorectal cancer by modulating mitochondrial membrane potential. Eur. J. Pharmacol..

[38] Li L., Xu T., Qi X. (2025). Balanced regulation of ROS production and inflammasome activation in preventing early development of colorectal cancer. Immunol. Rev..

[39] Lin S., Li Y., Zamyatnin A.A., Werner J., Bazhin A.V. (2018). Reactive oxygen species and colorectal cancer. J. Cell. Physiol..

[40] Xia T., Guo J., Zhang B., Song C., Zhao Q., Cui B., Liu Y. (2022). Bisphenol A Promotes the Progression of Colon Cancer Through Dual-Targeting of NADPH Oxidase and Mitochondrial Electron-Transport Chain to Produce ROS and Activating HIF-1α/VEGF/PI3K/AKT Axis. Front. Endocrinol..

[41] Jiang Y., Li Y., Wang Y., Li X. (2024). Furanodienone induces apoptosis via regulating the PRDX1/MAPKs/p53/caspases signaling axis through NOX4-derived mitochondrial ROS in colorectal cancer cells. Biochem. Pharmacol..

[42] Kuo T.T., Lin L.C., Chang H.Y., Chiang P.J., Wu H.Y., Chen T.Y., Hsia S.M., Huang T.C. (2022). Quantitative Proteome Analysis Reveals Melissa officinalis Extract Targets Mitochondrial Respiration in Colon Cancer Cells. Molecules.

[43] Lv C., Huang Y., Wang Q., Wang C., Hu H., Zhang H., Lu D., Jiang H., Shen R., Zhang W., Liu S. (2023). Ainsliadimer A induces ROS-mediated apoptosis in colorectal cancer cells via directly targeting peroxiredoxin 1 and 2. Cell Chem. Biol..

[44] Chan D.C. (2020). Mitochondrial Dynamics and Its Involvement in Disease. Annu. Rev. Pathol..

[45] Giacomello M., Pyakurel A., Glytsou C., Scorrano L. (2020). The cell biology of mitochondrial membrane dynamics. Nat. Rev. Mol. Cell Biol..

[46] Wu Z., Xiao C., Long J., Huang W., You F., Li X. (2024). Mitochondrial dynamics and colorectal cancer biology: mechanisms and potential targets. Cell Commun. Signal..

[47] Zhuo F.F., Li L., Liu T.T., Liang X.M., Yang Z., Zheng Y.Z., Luo Q.W., Lu J.H., Liu D., Zeng K.W., Tu P.F. (2023). Lycorine promotes IDH1 acetylation to induce mitochondrial dynamics imbalance in colorectal cancer cells. Cancer Lett..

[48] Wang S., Long H., Hou L., Feng B., Ma Z., Wu Y., Zeng Y., Cai J., Zhang D.W., Zhao G. (2023). The mitophagy pathway and its implications in human diseases. Signal Transduct. Target. Ther..

[49] Picca A., Faitg J., Auwerx J., Ferrucci L., D'Amico D. (2023). Mitophagy in human health, ageing and disease. Nat. Metab..

[50] Tang J., Peng W., Ji J., Peng C., Wang T., Yang P., Gu J., Feng Y., Jin K., Wang X., Sun Y. (2023). GPR176 Promotes Cancer Progression by Interacting with G Protein GNAS to Restrain Cell Mitophagy in Colorectal Cancer. Adv. Sci..

[51] Wu Q., Wang Z., Chen S., She X., Zhu S., Li P., Liu L., Zhao C., Li K., Liu A. (2024). USP26 promotes colorectal cancer tumorigenesis by restraining PRKN-mediated mitophagy. Oncogene.

[52] D'onofrio N., Martino E., Mele L. (2021). Colorectal Cancer Apoptosis Induced by Dietary δ-Valerobetaine Involves PINK1/Parkin Dependent-Mitophagy and SIRT3. Int. J. Mol. Sci..

[53] Yin K., Lee J., Liu Z., Kim H., Martin D.R., Wu D., Liu M., Xue X. (2021). Mitophagy protein PINK1 suppresses colon tumor growth by metabolic reprogramming via p53 activation and reducing acetyl-CoA production. Cell Death Differ..

[54] Kandettu A., Kuthethur R., Chakrabarty S. (2025). A detailed review on the role of miRNAs in mitochondrial-nuclear cross talk during cancer progression. Biochim. Biophys. Acta. Mol. Basis Dis..

[55] Abdelmaksoud N.M., Abulsoud A.I., Abdelghany T.M., Elshaer S.S., Rizk S.M., Senousy M.A. (2023). Mitochondrial remodeling in colorectal cancer initiation, progression, metastasis, and therapy: A review. Pathol. Res. Pract..

[56] Rainho M.d.A., Siqueira P.B., de Amorim Í.S.S., Mencalha A.L., Thole A.A. (2023). Mitochondria in colorectal cancer stem cells - a target in drug resistance. Cancer Drug Resist..

[57] Allocco A.L., Bertino F., Petrillo S., Chiabrando D., Riganti C., Bardelli A., Altruda F., Fiorito V., Tolosano E. (2022). Inhibition of Heme Export and/or Heme Synthesis Potentiates Metformin Anti-Proliferative Effect on Cancer Cell Lines. Cancers (Basel).

[58] Liu C., Liu Q., Yan A., Chang H., Ding Y., Tao J., Qiao C. (2020). Metformin revert insulin-induced oxaliplatin resistance by activating mitochondrial apoptosis pathway in human colon cancer HCT116 cells. Cancer Med..

[59] Sun J.G., Xiang J., Zeng X.L., Li X., Wu P., Fung K.P., Liu F.y. (2014). Clitocine induces apoptosis and enhances the lethality of ABT-737 in human colon cancer cells by disrupting the interaction of Mcl-1 and Bak. Cancer Lett..

[60] Maamer-Azzabi A., Ndozangue-Touriguine O., Bréard J. (2013). Metastatic SW620 colon cancer cells are primed for death when detached and can be sensitized to anoikis by the BH3-mimetic ABT-737. Cell Death Dis..

[61] Zhou Y., Zhang W., Wang B., Wang P., Li D., Cao T., Zhang D., Han H., Bai M., Wang X. (2024). Mitochondria-targeted photodynamic therapy triggers GSDME-mediated pyroptosis and sensitizes anti-PD-1 therapy in colorectal cancer. J. Immunother. Cancer.

[62] Fath M.A., Diers A.R., Aykin-Burns N., Simons A.L., Hua L., Spitz D.R. (2009). Mitochondrial electron transport chain blockers enhance 2-deoxy-D-glucose induced oxidative stress and cell killing in human colon carcinoma cells. Cancer Biol. Ther..

[63] Chen L., Hao M., Yan J., Sun L., Tai G., Cheng H., Zhou Y. (2021). Citrus-derived DHCP inhibits mitochondrial complex II to enhance TRAIL sensitivity via ROS-induced DR5 upregulation. J. Biol. Chem..

[64] Dong S., Zhang M., Cheng Z., Zhang X., Liang W., Li S., Li L., Xu Q., Song S., Liu Z. (2024). Redistribution of defective mitochondria-mediated dihydroorotate dehydrogenase imparts 5-fluorouracil resistance in colorectal cancer. Redox Biol..

[65] Huang C.R., Chu Y.T., Chang C.L., Yip H.K., Chen H.H. (2024). ZNF746 plays cardinal roles on colorectal cancer (CRC) cell invasion and migration and regulates mitochondrial dynamics and morphological changes of CRC cells-Role of combined melatonin and 5-FU regimen. J. Cell. Biochem..

[66] Bensard C.L., Wisidagama D.R., Olson K.A., Berg J.A., Krah N.M., Schell J.C., Nowinski S.M., Fogarty S., Bott A.J., Wei P. (2020). Regulation of Tumor Initiation by the Mitochondrial Pyruvate Carrier. Cell Metab..

[67] Li J., Wang Y., Shen W., Zhang Z., Su Z., Guo X., Pei P., Hu L., Liu T., Yang K., Guo L. (2024). Mitochondria-Modulating Liposomes Reverse Radio-Resistance for Colorectal Cancer. Adv. Sci..

[68] Tau S., Chamberlin M.D., Yang H., Marotti J.D., Muskus P.C., Roberts A.M., Carmichael M.M., Cressey L., Dragnev C.P.C., Demidenko E. (2025). Oxidative Phosphorylation Is a Metabolic Vulnerability of Endocrine Therapy-Tolerant Persister Cells in ER+ Breast Cancer. Cancer Res..

[69] Noonan A.M., Bunch K.P., Chen J.Q., Herrmann M.A., Lee J.M., Kohn E.C., O'Sullivan C.C., Jordan E., Houston N., Takebe N. (2016). Pharmacodynamic markers and clinical results from the phase 2 study of the SMAC mimetic birinapant in women with relapsed platinum-resistant or -refractory epithelial ovarian cancer. Cancer.

[70] Takahashi A., Hirohashi Y., Torigoe T., Tamura Y., Tsukahara T., Kanaseki T., Kochin V., Saijo H., Kubo T., Nakatsugawa M. (2013). Ectopically expressed variant form of sperm mitochondria-associated cysteine-rich protein augments tumorigenicity of the stem cell population of lung adenocarcinoma cells. PLoS One.

[71] Wang J., Zhang L., Xin H., Guo Y., Zhu B., Su L., Wang S., Zeng J., Chen Q., Deng R. (2022). Mitochondria-targeting folic acid-modified nanoplatform based on mesoporous carbon and a bioactive peptide for improved colorectal cancer treatment. Acta Biomater..

[72] Li Y., Liu J., Weichselbaum R.R., Lin W. (2024). Mitochondria-Targeted Multifunctional Nanoparticles Combine Cuproptosis and Programmed Cell Death-1 Downregulation for Cancer Immunotherapy. Adv. Sci..

[73] Mehmood T., Nasir Q., Younis I., Muanprasat C. (2025). Inhibition of Mitochondrial Dynamics by Mitochondrial Division Inhibitor-1 Suppresses Cell Migration and Metastatic Markers in Colorectal Cancer HCT116 Cells. J. Exp. Pharmacol..

[74] Faria A.V.S., Fonseca E.M.B., Fernandes-Oliveira P.d.S., de Lima T.I., Clerici S.P., Justo G.Z., Silveira L.R., Durán N., Ferreira-Halder C.V. (2022). Violacein switches off low molecular weight tyrosine phosphatase and rewires mitochondria in colorectal cancer cells. Bioorg. Chem..

[75] Shuwen H., Yinhang W., Jing Z., Qiang Y., Yizhen J., Quan Q., Yin J., Jiang L., Xi Y. (2023). Cholesterol induction in CD8(+) T cell exhaustion in colorectal cancer via the regulation of endoplasmic reticulum-mitochondria contact sites. Cancer Immunol. Immunother..

[76] Lu M., Sun L., Zhou J., Yang J. (2014). Dihydroartemisinin induces apoptosis in colorectal cancer cells through the mitochondria-dependent pathway. Tumour Biol..

[77] Rozman Antolikova N., Kello M., Zigova M., Tischlerova V., Petrilla V., Pirnik Z., Mojzisova G., Mojzis J. (2019). Naja ashei venom induces mitochondria-mediated apoptosis in human colorectal cancer cells. Acta Biochim. Pol..

[78] Liu M., Yang C., Peng X., Zheng S., He H., Wang W., Li Y. (2025). Formononetin suppresses colitis-associated colon cancer by targeting lipid synthesis and mTORC2/Akt signaling. Phytomedicine.

[79] Tang R., Zhang L., Lou J., Mo W., Zhao L., Li L., Zhang K., Yu Q. (2025). Formononetin prevents intestinal injury caused by radiotherapy in colorectal cancer mice via the Keap1-Nrf2 signaling pathway. Biochem. Biophys. Res. Commun..

[80] Delgado-Waldo I., Dokudovskaya S., Loissell-Baltazar Y.A., Pérez-Arteaga E., Coronel-Hernández J., Martínez-Vázquez M., Pérez-Yépez E.A., Lopez-Saavedra A., Jacobo-Herrera N., Pérez Plasencia C. (2024). Laherradurin Inhibits Colorectal Cancer Cell Growth by Induction of Mitochondrial Dysfunction and Autophagy Induction. Cells.

[81] Bamehr H., Saidijam M., Dastan D., Amini R., Pourjafar M., Najafi R. (2019). Ferula pseudalliacea induces apoptosis in human colorectal cancer HCT-116 cells via mitochondria-dependent pathway. Arch. Physiol. Biochem..

[82] Vichitsakul K., Laowichuwakonnukul K., Soontornworajit B., Poomipark N., Itharat A., Rotkrua P. (2023). Anti-proliferation and induction of mitochondria-mediated apoptosis by Garcinia hanburyi resin in colorectal cancer cells. Heliyon.

[83] Ryu H., Nam K.Y., Kim J.S., Hwang S.G., Song J.Y., Ahn J. (2018). The small molecule AU14022 promotes colorectal cancer cell death via p53-mediated G2/M-phase arrest and mitochondria-mediated apoptosis. J. Cell. Physiol..

[84] Liang B., Liu Z., Cao Y., Zhu C., Zuo Y., Huang L., Wen G., Shang N., Chen Y., Yue X. (2017). MC37, a new mono-carbonyl curcumin analog, induces G2/M cell cycle arrest and mitochondria-mediated apoptosis in human colorectal cancer cells. Eur. J. Pharmacol..

[85] Li T., Yuan G., Zhang L., Ye L., Li S., Fan Y., Sun J. (2015). ApoG2 inhibits the antiapoptotic protein, Mcl-1, and induces mitochondria-dependent apoptosis in human colorectal cancer cells. Mol. Med. Rep..

[86] Sun B.B., Fu L.N., Wang Y.Q., Gao Q.Y., Xu J., Cao Z.J., Chen Y.X., Fang J.Y. (2014). Silencing of JMJD2B induces cell apoptosis via mitochondria-mediated and death receptor-mediated pathway activation in colorectal cancer. J. Dig. Dis..

[87] Hsu H.C., Liu Y.S., Tseng K.C., Tan B.C.M., Chen S.J., Chen H.C. (2014). LGR5 regulates survival through mitochondria-mediated apoptosis and by targeting the Wnt/β-catenin signaling pathway in colorectal cancer cells. Cell. Signal..

[88] Li C., Zhang S., Zhu J., Huang W., Luo Y., Shi H., Yu D., Chen L., Song L., Yu R. (2022). A Novel Peptide Derived from Arca inflata Induces Apoptosis in Colorectal Cancer Cells through Mitochondria and the p38 MAPK Pathway. Mar. Drugs.

[89] Guo L.D., Chen X.J., Hu Y.H., Yu Z.J., Wang D., Liu J.Z. (2013). Curcumin inhibits proliferation and induces apoptosis of human colorectal cancer cells by activating the mitochondria apoptotic pathway. Phytother Res..

[90] Ma D., Tremblay P., Mahngar K., Akbari-Asl P., Collins J., Hudlicky T., McNulty J., Pandey S. (2012). A novel synthetic C-1 analogue of 7-deoxypancratistatin induces apoptosis in p53 positive and negative human colorectal cancer cells by targeting the mitochondria: enhancement of activity by tamoxifen. Invest. New Drugs.

[91] Nakagawa Y., Iinuma M., Naoe T., Nozawa Y., Akao Y. (2007). Characterized mechanism of alpha-mangostin-induced cell death: caspase-independent apoptosis with release of endonuclease-G from mitochondria and increased miR-143 expression in human colorectal cancer DLD-1 cells. Bioorg. Med. Chem..

[92] Bruno G., Pietrafesa M., Crispo F., Piscazzi A., Maddalena F., Giordano G., Conteduca V., Garofoli M., Porras A., Esposito F., Landriscina M. (2024). TRAP1 modulates mitochondrial biogenesis via PGC-1α/TFAM signalling pathway in colorectal cancer cells. J. Mol. Med..

[93] Zhang B., Liu Q., Wen W., Gao H., Wei W., Tang A., Qin B., Lyu H., Meng X., Li K. (2022). The chromatin remodeler CHD6 promotes colorectal cancer development by regulating TMEM65-mediated mitochondrial dynamics via EGF and Wnt signaling. Cell Discov..

[94] Žilinskas J., Stukas D., Jasukaitienė A. (2024). Assessing the Therapeutic Impacts of HAMLET and FOLFOX on BRAF-Mutated Colorectal Cancer: A Study of Cancer Cell Survival and Mitochondrial Dynamics In Vitro and Ex Vivo. Medicina (Kaunas).

[95] Wang S., Jiang X., Xie X., Yin J., Zhang J., Liu T., Chen S., Wang Y., Zhou X., Wang Y. (2022). piR-823 inhibits cell apoptosis via modulating mitophagy by binding to PINK1 in colorectal cancer. Cell Death Dis..

[96] Yan C., Luo L., Guo C.Y., Goto S., Urata Y., Shao J.H., Li T.S. (2017). Doxorubicin-induced mitophagy contributes to drug resistance in cancer stem cells from HCT8 human colorectal cancer cells. Cancer Lett..

[97] Wang Z., Yu C., Xie G., Tao K., Yin Z., Lv Q. (2025). USP14 inhibits mitophagy and promotes tumorigenesis and chemosensitivity through deubiquitinating BAG4 in microsatellite instability-high colorectal cancer. Mol. Med..

[98] Liang L., Sun W., Wei X., Wang L., Ruan H., Zhang J., Li S., Zhao B., Li M., Cai Z., Huang J. (2023). Oxymatrine suppresses colorectal cancer progression by inhibiting NLRP3 inflammasome activation through mitophagy induction in vitro and in vivo. Phytother Res..

[99] Zhang C., Zeng C., Xiong S., Zhao Z., Wu G. (2022). A mitophagy-related gene signature associated with prognosis and immune microenvironment in colorectal cancer. Sci. Rep..

[100] Weng J.S., Huang J.P., Yu W., Xiao J., Lin F., Lin K.N., Zang W.D., Ye Y., Lin J.P. (2023). Mitophagy-related gene signature predicts prognosis, immune infiltration and chemotherapy sensitivity in colorectal cancer. World J. Gastrointest. Oncol..

[101] Zhang X., Meng L., Zu T., Zhou Q. (2025). Identification of necroptosis & mitophagy-related key genes and their prognostic value in colorectal cancer. Discov. Oncol..

[102] Gao H., Zou Q., Ma L., Cai K., Sun Y., Lu L., Ren D., Hu B. (2023). Unveiling mitophagy-mediated molecular heterogeneity and development of a risk signature model for colorectal cancer by integrated scRNA-seq and bulk RNA-seq analysis. Gastroenterol. Rep..

[103] Tao Z.H., Han J.X., Xu J., Zhao E., Wang M., Wang Z., Lin X.L., Xiao X.Y., Hong J., Chen H. (2025). Screening of patient-derived organoids identifies mitophagy as a cell-intrinsic vulnerability in colorectal cancer during statin treatment. Cell Rep. Med..

[104] Xu Z., Zhao G., Zhang L., Qiao C., Wang H., Wei H., Liu R., Liu P., Zhang Y., Zhu W., You W. (2024). Tong-Xie-Yao-Fang induces mitophagy in colonic epithelial cells to inhibit colitis-associated colorectal cancer. J. Ethnopharmacol..

[105] Wang H., Ji Y., Deng S., Qin X.Y., Ye X.T., Sun Y.Y., Che X.Y., Yang L., Huang C.Y., Chen Y., Liu Y.P. (2025). SQYC formula improves the efficacy of PD-1 monoclonal antibodies in MSS colorectal cancer by regulating dendritic cell mitophagy via the PINK1-Parkin pathway. Phytomedicine.

[106] Donisi I., Balestrieri A., Del Vecchio V., Bifulco G., Balestrieri M.L., Campanile G., D'Onofrio N. (2025). l-Carnitine and Acetyl-l-Carnitine Induce Metabolism Alteration and Mitophagy-Related Cell Death in Colorectal Cancer Cells. Nutrients.

[107] Yang S.T., Fan J.B., Liu T.T., Ning S., Xu J.H., Zhou Y.J., Deng X. (2022). Development of Strigolactones as Novel Autophagy/Mitophagy Inhibitors against Colorectal Cancer Cells by Blocking the Autophagosome-Lysosome Fusion. J. Med. Chem..

[108] Borcherding N., Brestoff J.R. (2023). The power and potential of mitochondria transfer. Nature.

[109] Saha T., Dash C., Jayabalan R., Khiste S., Kulkarni A., Kurmi K., Mondal J., Majumder P.K., Bardia A., Jang H.L., Sengupta S. (2022). Intercellular nanotubes mediate mitochondrial trafficking between cancer and immune cells. Nat. Nanotechnol..

[110] Sasaki R., Luo Y., Kishi S., Ogata R., Nishiguchi Y., Sasaki T., Ohmori H., Fujiwara-Tani R., Kuniyasu H. (2025). Oxidative High Mobility Group Box-1 Accelerates Mitochondrial Transfer from Mesenchymal Stem Cells to Colorectal Cancer Cells Providing Cancer Cell Stemness. Int. J. Mol. Sci..

[111] Frisbie L., Pressimone C., Dyer E., Baruwal R., Garcia G., St Croix C., Watkins S., Calderone M., Gorecki G., Javed Z. (2024). Carcinoma-associated mesenchymal stem cells promote ovarian cancer heterogeneity and metastasis through mitochondrial transfer. Cell Rep..

[112] Goliwas K.F., Libring S., Berestesky E., Gholizadeh S., Schwager S.C., Frost A.R., Gaborski T.R., Zhang J., Reinhart-King C.A. (2023). Mitochondrial transfer from cancer-associated fibroblasts increases migration in aggressive breast cancer. J. Cell Sci..

[113] Del Vecchio V., Rehman A., Panda S.K., Torsiello M., Marigliano M., Nicoletti M.M., Ferraro G.A., De Falco V., Lappano R., Lieto E. (2024). Mitochondrial transfer from Adipose stem cells to breast cancer cells drives multi-drug resistance. J. Exp. Clin. Cancer Res..

[114] Hoover G., Gilbert S., Curley O., Obellianne C., Lin M.T., Hixson W., Pierce T.W., Andrews J.F., Alexeyev M.F., Ding Y. (2025). Nerve-to-cancer transfer of mitochondria during cancer metastasis. Nature.

[115] He X., Zhong L., Wang N., Zhao B., Wang Y., Wu X., Zheng C., Ruan Y., Hou J., Luo Y. (2024). Gastric Cancer Actively Remodels Mechanical Microenvironment to Promote Chemotherapy Resistance via MSCs-Mediated Mitochondrial Transfer. Adv. Sci..

[116] Ippolito L., Morandi A., Taddei M.L., Parri M., Comito G., Iscaro A., Raspollini M.R., Magherini F., Rapizzi E., Masquelier J. (2019). Cancer-associated fibroblasts promote prostate cancer malignancy via metabolic rewiring and mitochondrial transfer. Oncogene.

[117] Bjerring J.S., Khodour Y., Peterson E.A., Sachs P.C., Bruno R.D. (2025). Intercellular mitochondrial transfer contributes to microenvironmental redirection of cancer cell fate. FEBS J..

[118] Jing M., Xiong X., Mao X., Song Q., Zhang L., Ouyang Y., Pang Y., Fu Y., Yan W. (2024). HMGB1 promotes mitochondrial transfer between hepatocellular carcinoma cells through RHOT1 and RAC1 under hypoxia. Cell Death Dis..

[119] Haupts A., Vogel A., Foersch S., Hartmann M., Maderer A., Wachter N., Huber T., Kneist W., Roth W., Lang H. (2021). Comparative analysis of nuclear and mitochondrial DNA from tissue and liquid biopsies of colorectal cancer patients. Sci. Rep..

[120] Qu F., Chen Y., Wang X., He X., Ren T., Huang Q., Zhang J., Liu X., Guo X., Gu J., Xing J. (2015). Leukocyte mitochondrial DNA content: a novel biomarker associated with prognosis and therapeutic outcome in colorectal cancer. Carcinogenesis.

[121] Dieli-Conwright C.M., Sami N., Norris M.K., Wan J., Kumagai H., Kim S.J., Cohen P. (2021). Effect of aerobic and resistance exercise on the mitochondrial peptide MOTS-c in Hispanic and Non-Hispanic White breast cancer survivors. Sci. Rep..

[122] West M.A., Loughney L., Lythgoe D., Barben C.P., Adams V.L., Bimson W.E., Grocott M.P.W., Jack S., Kemp G.J. (2014). The effect of neoadjuvant chemoradiotherapy on whole-body physical fitness and skeletal muscle mitochondrial oxidative phosphorylation in vivo in locally advanced rectal cancer patients--an observational pilot study. PLoS One.

[123] Mijwel S., Cardinale D.A., Norrbom J., Chapman M., Ivarsson N., Wengström Y., Sundberg C.J., Rundqvist H. (2018). Exercise training during chemotherapy preserves skeletal muscle fiber area, capillarization, and mitochondrial content in patients with breast cancer. FASEB J.

[124] Feng L.R., Wolff B.S., Liwang J., Regan J.M., Alshawi S., Raheem S., Saligan L.N. (2020). Cancer-related fatigue during combined treatment of androgen deprivation therapy and radiotherapy is associated with mitochondrial dysfunction. Int. J. Mol. Med..

[125] Janku F., Lorusso P., Mansfield A.S., Nanda R., Spira A., Wang T., Melhem-Bertrandt A., Sugg J., Ball H.A. (2021). First-in-human evaluation of the novel mitochondrial complex I inhibitor ASP4132 for treatment of cancer. Invest. New Drugs.

[126] Nicolson G.L. (2005). Lipid replacement/antioxidant therapy as an adjunct supplement to reduce the adverse effects of cancer therapy and restore mitochondrial function. Pathol. Oncol. Res..

[127] Moore A., Lan Q., Hofmann J.N., Liu C.S., Cheng W.L., Lin T.T., Berndt S.I. (2017). A prospective study of mitochondrial DNA copy number and the risk of prostate cancer. Cancer Causes Control..

